# Determination of Odour Interactions of Three-Component Gas Mixtures Using an Electronic Nose

**DOI:** 10.3390/s17102380

**Published:** 2017-10-18

**Authors:** Bartosz Szulczyński, Jacek Namieśnik, Jacek Gębicki

**Affiliations:** 1 Department of Chemical and Process Engineering, Faculty of Chemistry, Gdansk University of Technology, 11/12 G. Narutowicza Str., 80233 Gdańsk, Poland; bartosz.szulczynski@pg.gda.pl; 2 Department of Analytical Chemistry, Faculty of Chemistry, Gdansk University of Technology, 11/12 G. Narutowicza Str., 80233 Gdańsk, Poland; jacek.namiesnik@pg.edu.pl

**Keywords:** electronic nose, odour interactions, principal component regression, odour intensity, hedonic tone

## Abstract

The paper presents an application of an electronic nose prototype comprised of six TGS-type sensors and one PID-type sensor to identify odour interaction phenomena in odorous three-component mixtures. The investigation encompassed eight odorous mixtures—toluene-acetone-triethylamine and formaldehyde-butyric acid-pinene—characterized by different odour intensity and hedonic tone. A principal component regression (PCR) calibration model was used for evaluation of predicted odour intensity and hedonic tone. Correctness of identification of odour interactions in the odorous three-component mixtures was determined based on the results obtained with the electronic nose. The results indicated a level of 75–80% for odour intensity and 57–73% for hedonic tone. The average root mean square error of prediction amounted to 0.03–0.06 for odour intensity determination and 0.07–0.34 for hedonic tone evaluation of the odorous three-component mixtures.

## 1. Introduction

The capability to identify and discriminate between odorous substances is largely limited to methods and instruments based on the sense of smell. Progress in science and technology has increased interest in devices designed and operating analogously to human senses. The last 30 years have witnessed the elaboration of analytical systems capable of replacing odour evaluation by humans, at least to certain extent [1,2,3,4,5,6,7,8,9]. The instruments have that attracted attention due to the utilization of a wide range of chemical sensors are electronic noses. These devices, being analogues of the human sense of smell, can be applied in many fields of science and industry such as medical diagnostics [10,11,12,13], environmental protection [14,15,16,17,18,19,20,21], food and agricultural industries [22,23,24,25], and forensic science and criminology [26,27,28,29]. Electronic nose instruments allow holistic analysis of gas mixture compositions without the need to separate and identify of particular components [30,31], moreover, they have gained increasing popularity as far as measurement of the gas mixtures with very low concentration levels of their particular components is concerned [32,33]. These devices are characterized by additional advantages as compared to other techniques for odour analysis such as olfactometry or gas chromatography. As opposed to olfactometric techniques there is no need for olfactory adaptation and trained personnel for defined odour perception. Electronic noses offer rapid analysis and lower costs than chromatographic techniques. With all their advantages and certain limitations electronic nose instruments are complementary with respect to the aforementioned odour analysis measurement techniques.

The sensation of odour is relatively difficult to describe quantitatively. There are four basic features of odour determined during investigations and description of odorous compounds: odour concentration, intensity, hedonic tone and olfactory threshold. In the case of gas mixtures containing odorous compounds there is a discrepancy between sensed odour and summary odour (being a sum of the odours of particular components) [34,35,36].

Knowledge about olfactory thresholds of pure chemical compounds does not allow anticipation of the odour of their mixtures with other compounds. The sensible range of the mixtures is not an additive quantity. This is a result of so-called odour interactions consisting in mutual masking, amplification or attenuation of odours. The investigations on odour interaction types have been carried out for many years, however no satisfactory explanation for the mechanism of these processes has been provided so far. Most frequently the objects under investigation are air samples containing no more than two or three odorant types [37,38,39]. Dependence between physical stimuli acting on senses and psychical feelings is described by a field of knowledge called psychophysics. In the case of interactions models of odour interaction are created, which describe the dependence between odour intensity of air containing pollution mixtures and:

- odour intensity, which would be caused by the mixture components if they were present separately (perception models),
- concentrations of mixture components and their psychophysical characteristics (psychophysical models).

None of numerous elaborated models possesses a general character. The perception models of interaction combine odour intensity of a mixture (*I_AB_*) with intensity of its components when present separately (*I_A_*, *I_B_*). The best-known empirical equation combining odour intensity of a mixture with odour intensity of the individual components is Equation (1), the Zwaardemaker equation (from 1908) often called vector summation of intensities:

(1) $$I_{AB}^{2}=I_{A}^{2}+I_{B}^{2}+2I_{A}I_{B}cos\alpha_{AB}$$

The interaction coefficient (a) present in the equation is approximately constant for one pair of mixture components. Recently the investigations on determination of this coefficient for the compounds from aldehyde, ester or aromatic hydrocarbons groups were conducted by Yan et al. who determined its values at the level of: −0.22 for aldehydes, −0.25 for esters, −0.129 for aromatic hydrocarbons [40,41]. Generally, the odour interaction coefficients falls between −0.326 and −0.423, although there were also cases when it took lower values from −0.156 to −0.208. A couple of other perception models have been elaborated, including the Berglund, Patte and Laffort or U models [42,43,44,45].

Odour intensity depends on the number of odorous substance molecules, which are in contact with olfactory receptors, namely on odorous substance concentration in the inhaled air. Intensity is defined as “strength of odour perception”, which is triggered by particular olfactory stimulus. Most frequently verbal point scales or reference scales are used in order to determine odour intensity. An example of the verbal scale can be a 6-grade scale recommended in the German guidelines VDI 3940, where 0 value means no odour and 6 value describes an extremely strong odour.

As far as odour hedonic tone is concerned, hedonic interaction of odour is an interaction of an odorous substance, which, upon evaluation of a given olfactory sensation, is attributed to a certain feature located between two extreme situations described as extremely pleasant and extremely unpleasant, respectively. In practice odour hedonic tone is evaluated in the way similar to odour intensity using single-dimensional scales (verbal, graphical, point ones). Negative values of the verbal scale describe unpleasant olfactory sensations, positive values of that scale correspond to pleasant sensations, a value of 0 is associated with a neutral olfactory sensation.

Determination of odour intensity or odour hedonic tone using an electronic nose requires application of a “teaching under supervision” approach. Depending on the research problem these techniques (teaching under supervision) are employed to construct calibration, discrimination or classification models. Construction of the above models utilizes a set of explanatory variables (signals from the sensors comprising an electronic nose) and a set of dependent variables (values of odour intensity or hedonic tone expressed on the verbal scale). A calibration method task is construction of a model, which allows quantitative evaluation of particular property or the properties based on the set of explanatory variables. The most popular calibration techniques include multiple linear regression (MLR), principal component regression (PCR) and partial least squares (PLS). Operation on a big number of correlated variables results in limited applicability of the MLR models. Hence, the main methods used for construction of linear calibration models are PCR and PLS, as they are capable of managing correlated variables. These methods have found successful application for monitoring of odour concentration changes in processes such as biofiltration or sewage treatment as well as a support to dynamic olfactory measurements [46,47,48,49,50,51,52,53,54]. Literature in the field [55] reveals that for binary mixtures (*n*-butanol, acetone) of odorous compounds, a linear regression between odour intensity and averaged sensor response is appropriate to represent the relationship between odour intensity and electronic nose measurement when conducting polymer sensors are used, but it is inadequate for metal oxide sensors. Moreover, for binary mixtures of odorous compounds (*n*-butanol, acetone), a neural network can be trained to accurately predict odour intensity from commercial electronic nose sensor responses.

This paper describes an attempt to apply the PCR method and an electronic nose instrument to determine odour interactions of three-component gas mixtures characterized by different types of odour, which are components of the odorous mixtures typically present in municipal landfills or sewage treatment plants. The PCR method was used to determine if there occurred amplification of odour intensity with respect to the theoretical value estimated with Patte and Laffort model. The same method was utilized to check whether the electronic nose could be used to predict odour hedonic tone and how this value differed from the theoretical value calculated as algebraic sum of particular odorous components. Proposed PCR models were verified via coefficient of determination (R^2^) and root mean square error of prediction (RMSEP).

## 2. Materials and Methods 

### 2.1. Types of Three-Component Mixtures and Their Preparation

The investigation employed pure substances and three-component mixtures of the following compounds: toluene, acetone, triethylamine (set A and B) and formaldehyde, butyric acid, α-pinene (set C and D). Table 1 presents characteristics of the investigated odorous compounds including odour type, vapour pressure, and olfactory threshold in the gas phase [56,57,58]. 

Five aqueous solutions characterized by a 2-step dilution were prepared for each of the investigated substances. For acetone and formaldehyde the concentrations were as follows: 200, 400, 800, 1600, 3200 ppm v/v in deionised water. For toluene, triethylamine, butyric acid the concentrations formed a series: 5, 10, 20, 40, 80 ppm v/v in deionised water. In case of pinene the concentrations were as follows: 1, 2, 4, 8, 16 ppm v/v in deionised water. Prepared solutions were used for determination of odour intensity and hedonic tone of each sample, which was performed by a team of assessors. Obtained results were utilized to plot odour intensity versus logarithm of concentration of particular odorous substance in water as well as to depict hedonic tone versus logarithm of concentration of particular odorous substance in water. These plots allowed estimation of the olfactory thresholds of given substances in aqueous solution in order to confront them with the theoretical values. Additionally, the plots were used to determine concentration of particular substances in deionised water corresponding to odour intensities equal to 1 and 2 according to the scale proposed in the VDI 3940 guidelines. 

### 2.2. Olfactory Triangles

**Figure 1:**
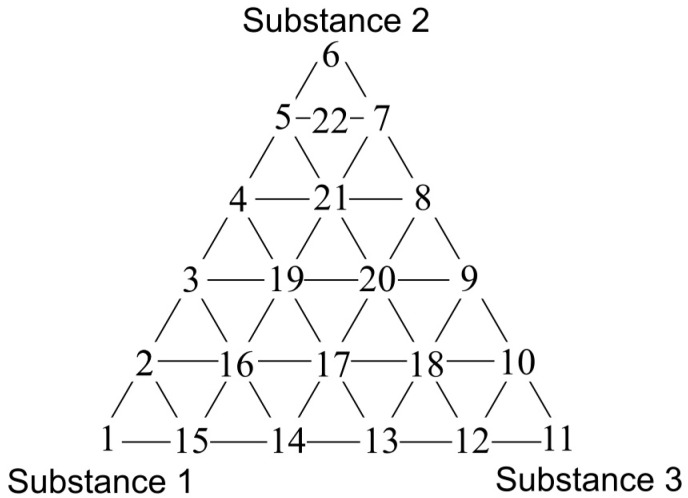
Triangle presenting distribution of three-component mixtures samples.

A triangle presenting distribution of three-component mixture samples is shown in Figure 1. The samples present on the vertexes of the triangle (1, 6 and 11) are comprised of pure substances in deionised water, characterized by an odour intensity equal to 1 or an odour intensity equal to 2. The points located on the sides of the triangle represent two-component mixtures of the respective compounds in deionised water. Three-component mixtures in deionised water are inside the triangle. The composition of prepared three-component mixtures (set A and B with intensity 1 and 2; set C and D with intensity 1 and 2) is shown in Table 2. Moreover, Table 2 contains the information about hedonic tone of particular samples calculated based on the plots described in the Section 2.1.

### 2.3. Measurement of Odour Intensity and Hedonic Tone 

The investigation was carried out by a group of assessors whose task was the olfactory evaluation of the prepared samples. This group consisted of five persons trained according to St. Croix Sensory 2006, a procedure elaborated by St. Croix Sensory, Inc. (Stillwater, MN, USA). The assessors were also aware of and followed the rules concerning olfactory investigations contained in the standard PN-EN 13 725 “Air quality. Determination of odour concentration by dynamic olfactometry”. The task of each assessor was determination of odour intensity and hedonic tone of prepared samples of aqueous solutions. Each sample was attributed the odour intensity within the range from 0 to 6 and the hedonic tone from −4 to 4. The assessors evaluated the total of 880 samples in case of the interactions within the olfactory triangle and 450 samples in case of the functional dependences: odour intensity versus logarithm of concentration of particular odorous substance in water as well as hedonic tone versus logarithm of concentration of particular odorous substance in water. 

### 2.4. Description of Experimental Setup for Electronic Nose Investigations

**Figure 2:**
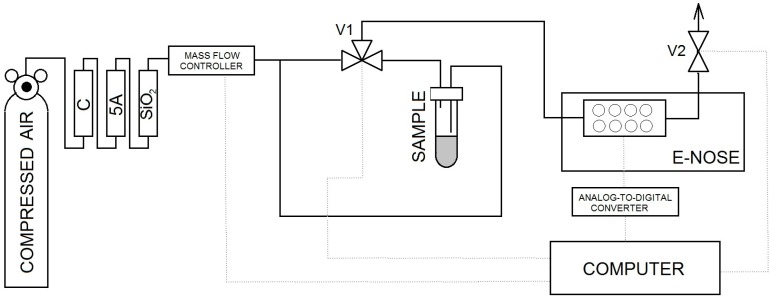
Scheme of experimental setup containing electronic nose.

A scheme of the experimental setup is illustrated in Figure 2. It consisted of:

- bottle with carrier gas (compressed air) with reducing valve,
- system of air purification containing three filters filled successively with: active carbon (C), molecular sieve 5A and silica (SiO_2_),
- three-way V1and cut-off V2 valves,
- sample mounting system,
- mass flow controller (red-y smart series GSC-B9SS-BB23, Voegtlin, Aesch, Switzerland)
- prototype of electronic nose equipped with a matrix of seven sensors: six sensors of MOS-type (TGS 813,TGS 816, TGS 822, TGS 2444, TGS 2602, TGS 2620-FIGARO USA Inc., Arlington Heights, IL, USA) and one PID-type sensor (MiniPID-Ion Science Ltd., Cambridge, UK)
- PC-class computer.

### 2.5. Methodology of Measurement Using Electronic Nose

Clean air was flowing through a measurement system. Inlet and outlet tubes were connected to the investigated sample in order to provide carrier gas flow. After mounting of a tube with the sample a three-way valve V1 was switched in order to change a direction of air flow. Air was supplied into the sample via the inlet tube while the outlet tube led aerated phase into a measurement sensors chamber of the electronic nose. Recording of signals started 25 s after the moment when air had been passed through the probes with investigated substances. The signal was recorded for 15 s. After that time the three-way valve was switched into its initial position enabling cleaning of the measurement system due to undisturbed flow of the carrier gas until e-nose signal returned to the initial level. The measurement parameters were determined via optimization method and they were as follows:

- volumetric flow rate of air, determined using the rotameter, was equal 0.3 L/min,
- time of carrier gas flow through the sample: 25 s,
- signal recording: 15 s.

The system operated in a stop-flow mode meaning: 25 s of carrier gas flow, 15 s of carrier gas flow interruption, 5 min of carrier gas flow. A total of 880 samples were investigated in order to define odour interactions in the olfactory triangle, wheminutere 440 samples were the training ones and 440 samples constituted the tested ones. In case of utilization of the semiconductor sensors (in the investigations presented) a quotient technique of baseline correction was applied:

(2) $$S_{meas}=\frac{S\left(t\right)}{S_{0}}$$

where: *S_meas_*–value of signal after baseline correction, *S*(*t*)–value of signal at given time instant prior to baseline correction, *S*_0_–value of baseline signal.

This correction allowed reduction of the drift exhibiting multiplicative character. Application of this technique was found justified and purposeful due to its theoretical background, which suggests that the quotient method should provide the best effects [59]. 

### 2.6. Data Analysis

Analysis of the data obtained with the electronic nose prototype was carried out using free R software being a part of Free Software Foundation (Free Software Foundation, Boston, MA, USA). All original variables were subjected to transformation via autoscaling, which resulted in variance of all properties equal each other and equal 1.

## 3. Results and Discussion

### 3.1. Determination of Olfactory Thresholds for Particular Odorous Compounds in Aqueous Solution

**Figure 3:**
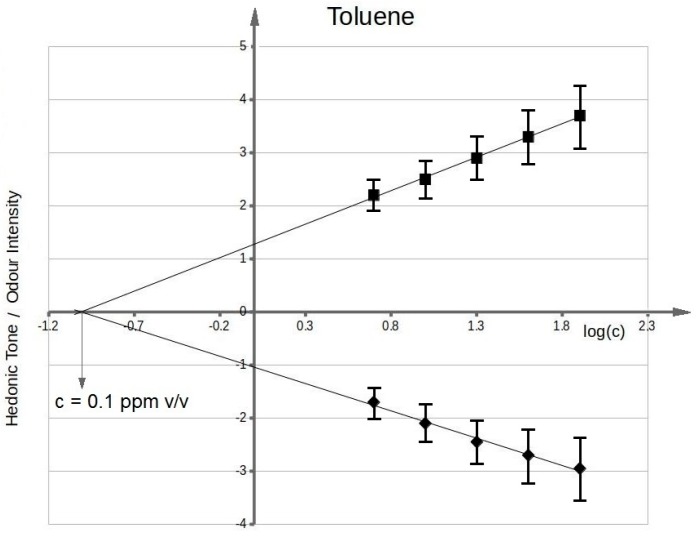
Mean odour intensity versus logarithmic concentration of toluene in deionized water and mean hedonic tone versus logarithmic concentration of toluene in deionized water.

**Figure 4:**
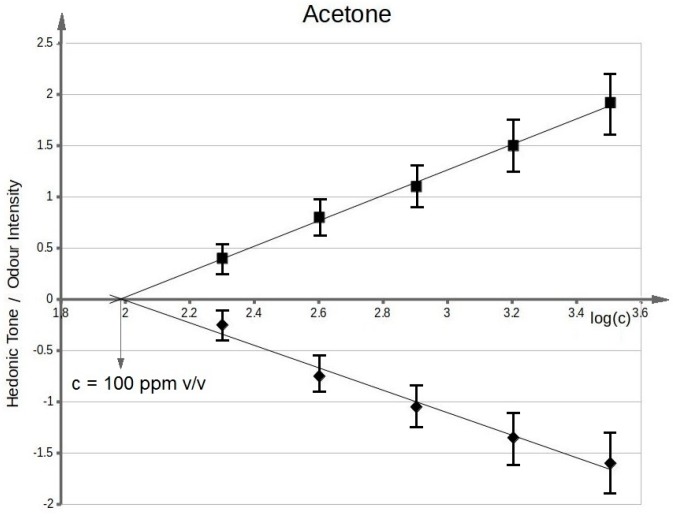
Mean odour intensity versus logarithmic concentration of acetone in deionized water and mean hedonic tone versus logarithmic concentration of acetone in deionized water.

**Figure 5:**
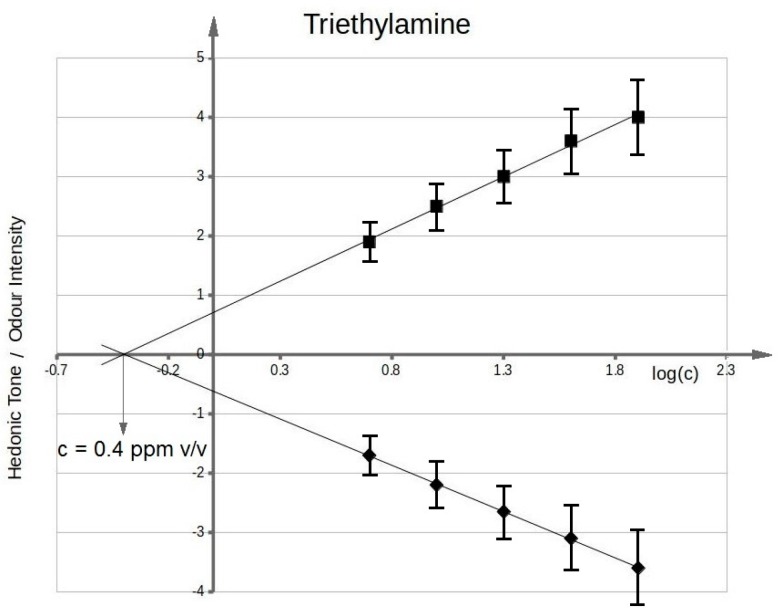
Mean odour intensity versus logarithmic concentration of triethylamine in deionized water and mean hedonic tone versus logarithmic concentration of triethylamine in deionized water.

**Figure 6:**
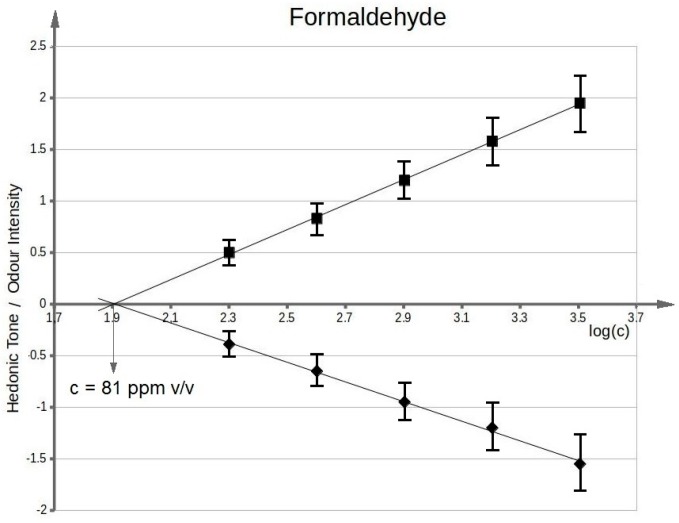
Mean odour intensity versus logarithmic concentration of formaldehyde in deionized water and mean hedonic tone versus logarithmic concentration of formaldehyde in deionized water.

**Figure 7:**
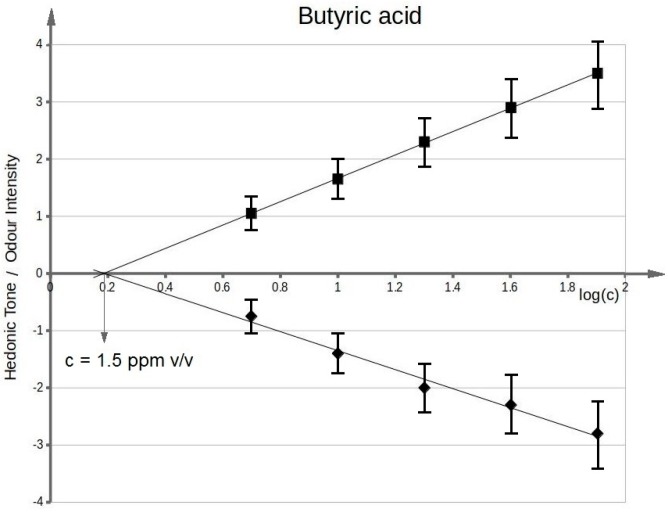
Mean odour intensity versus logarithmic concentration of butyric acid in deionized water and mean hedonic tone versus logarithmic concentration of butyric acid in deionized water.

**Figure 8:**
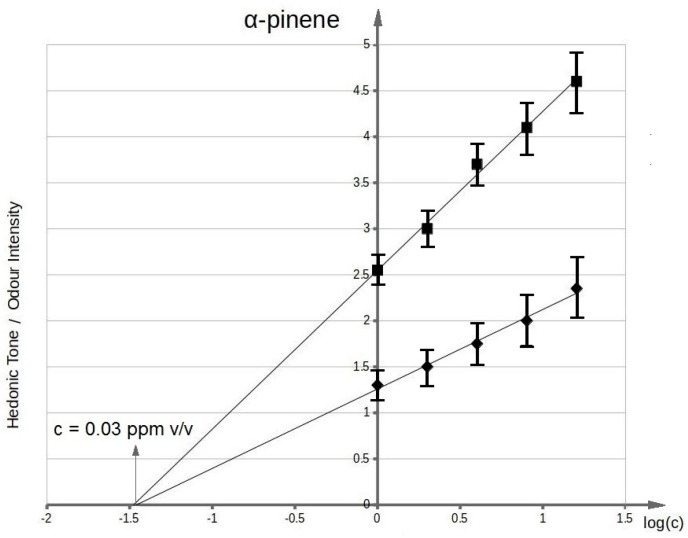
Mean odour intensity versus logarithmic concentration of α-pinene in deionized water and mean hedonic tone versus logarithmic concentration of α-pinene in deionized water.

Figure 3 presents functional dependency between odour intensity of toluene aqueous solutions and logarithm of toluene concentration in deionized water. It can be seen that the value of olfactory threshold equals 0.1 ppm v/v. The characteristics (Figure 3) illustrating dependency between hedonic tone and logarithm of toluene concentration in deionized water also intersect the axis of abscissae in the point where no odour can be sensed. This value is equal to olfactory threshold value within the limits of random error. Analogous procedure was applied in order to evaluate olfactory thresholds for the remaining samples of odorous compounds in aqueous solutions. Figure 4 shows functional dependency of odour intensity and hedonic tone versus logarithm of acetone concentration in deionized water. Figure 5 presents the functional dependency of odour intensity and hedonic tone versus logarithm of triethylamine concentration in deionized water. The remaining Figure 6, Figure 7 and Figure 8 illustrate functional dependency of odour intensity and hedonic tone versus logarithm of formaldehyde (Figure 6), butyric acid (Figure 7), α-pinene (Figure 8) concentration. The values of olfactory threshold obtained based on the determined functional dependencies are gathered in Table 3.

The Table 3 also includes the theoretical values of olfactory thresholds in aqueous solutions. It can be seen that the obtained experimental values (in the range of random error) are within the limits of olfactory thresholds reported in literature.

The obtained function dependencies were used to determine concentration of particular odorous compounds, for which odour intensity was equal to 1 and 2. The plots of hedonic tone versus logarithm of concentration of a given compound in deionized water were utilized to assess the values of hedonic tone, at which odour intensity equalled 1 and 2. Analogous approach was employed for determination of the remaining values of odour intensity and hedonic tone where samples concentrations constituted 80%, 60%, 40%, 20%, 10% of the concentration, at which value of odour intensity was 1 or 2. In this way 22 samples were prepared, which contributed to a sample distribution in the olfactory triangle of three-component mixture (set A and B—toluene, acetone, triethylamine of odour intensity ca. 1 and ca. 2; set C and D—formaldehyde, butyric acid, pinene of odour intensity ca. 1 and ca. 2).

### 3.2. Determination of Theoretical Values of Odour Intensity and Hedonic Tone of Three-Component Mixtures Using Perception Model

In case of determination of the theoretical odour intensity of 22 samples of three-component mixtures for A, B, C, D sets the Patte and Laffort model was used, the mathematical form of which is presented below Equation (3):

(3) $$I_{mix1,2,3}=\sqrt{I_{1}^{2}+I_{2}^{2}+I_{3}^{2}}$$

Evaluation of the theoretical hedonic tone of 22 samples of three-component mixtures for A, B, C, D sets involved algebraic summation, the mathematical form of which is shown below Equation (4):

(4) $$HT_{mix1,2,3}=\overset{3}{\underset{i=1}{\sum }}HT_{i}$$

In this way a distribution of the theoretical values of odour intensity and hedonic tone of 22 samples contributing to odour variation within the olfactory triangle was found for four investigated measurement sets.

Independently of determined theoretical values a group of assessors conducted an investigation aimed at evaluation of odour intensity and hedonic tone of 22 samples belonging to four sets of examined three-component mixtures. It enabled identification of the measurement points (samples) within the olfactory triangle where odour interaction occurred. A statistical U Manna-Whitney test was utilized to find the measurement points within the olfactory triangle, which differed statistically from the theoretical values. 

### 3.3. Determination of Measurement Points Within Olfactory Triangle Where Odour Interaction Phenomenon Was Discovered by A Group of Assessors

**Figure 9:**
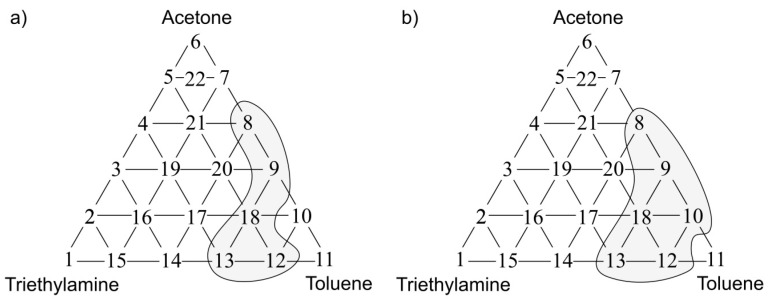
(**a**) Olfactory triangle for toluene-acetone-triethylamine mixture (set A) with indicated places of odour intensity amplification; (**b**) with indicated places of hedonic tone amplification. Sensory analysis.

The statistical test allowed identification of the places within the olfactory triangle where the values of odour intensity and hedonic tone exhibited significant statistical difference from the theoretical values determined according to a procedure described in the Section 3.2. Figure 9a presents the olfactory triangle for the toluene-acetone-triethylamine mixture (where odour intensity of the samples 1, 6, 11 equalled 1, set A) where one can observe five measurement points revealing amplification of odour intensity, namely odour synergism phenomenon. Figure 9b shows the olfactory triangle for the same three-component mixture, in which six measurement points characterized by amplification of hedonic tone are evident (set A).

**Figure 10:**
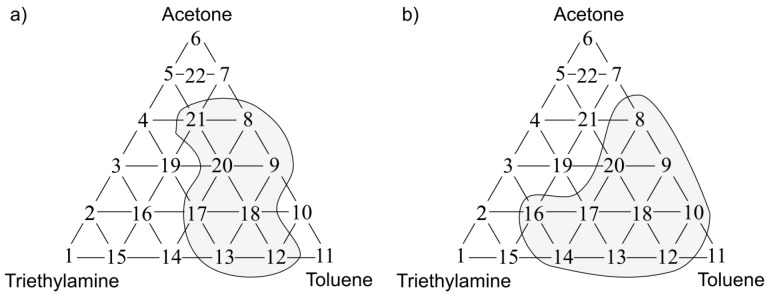
(**a**) Olfactory triangle for toluene-acetone-triethylamine mixture (set B) with indicated places of odour intensity amplification; (**b**) with indicated places of hedonic tone amplification. Sensory analysis.

**Figure 11:**
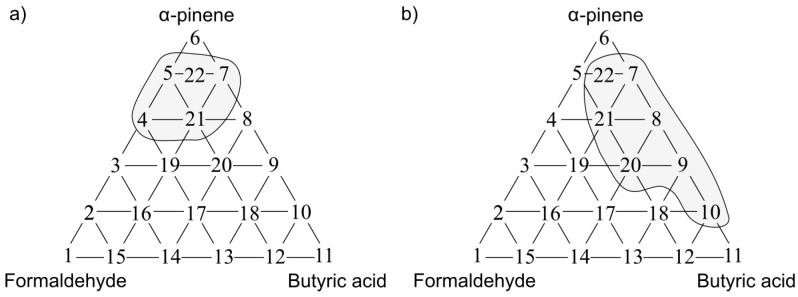
(**a**) Olfactory triangle for formaldehyde-butyric acid-pinene mixture (set C) with indicated places of odour intensity amplification; (**b**) with indicated places of hedonic tone amplification.

**Figure 12:**
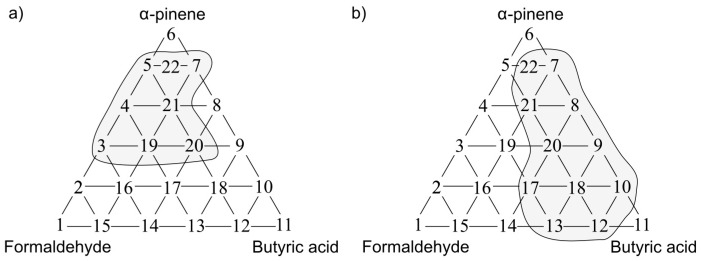
(**a**) Olfactory triangle for formaldehyde-butyric acid-pinene mixture (set D) with indicated places of odour intensity amplification; (**b**) with indicated places of hedonic tone amplification.

Analogous olfactory triangles with the places of odour intensity and hedonic tone amplification are illustrated in Figure 10, Figure 11 and Figure 12. In Figure 10a one can see the olfactory triangle for the toluene-acetone-triethylamine mixture (where odour intensity of the samples 1, 6, 11 equalled 2, set B) where 8 measurement points revealed amplification of odour intensity. Figure 10b depicts the olfactory triangle for the same three-component mixture, in which 10 measurement points are characterized by amplification of hedonic tone.

Figure 11a presents the olfactory triangle for the formaldehyde-butyric acid-pinene mixture (where odour intensity of the samples 1, 6, 11 equalled 1, set C) where one can observe five measurement points revealing amplification of odour intensity. Figure 11b shows the olfactory triangle for the same three-component mixture, in which seven measurement points characterized by amplification of hedonic tone are evident (set C).

In Figure 12a one can notice the olfactory triangle for the formaldehyde-butyric acid-pinene mixture (where odour intensity of the samples 1, 6, 11 equalled 2, set D) where eight measurement points revealed amplification of odour intensity. Figure 12b depicts the olfactory triangle for the same three-component mixture, in which 11 measurement points are characterized by amplification of hedonic tone (set D). Generally, it can be observed that an increase in odour intensity or hedonic tone is accompanied of an increase in the number of samples, which are characterized by odour amplification phenomenon.

### 3.4. Determination of Measurement Points within Olfactory Triangle Where Odour Interaction Phenomenon Was Discovered with Electronic Nose Instrument

**Figure 13:**
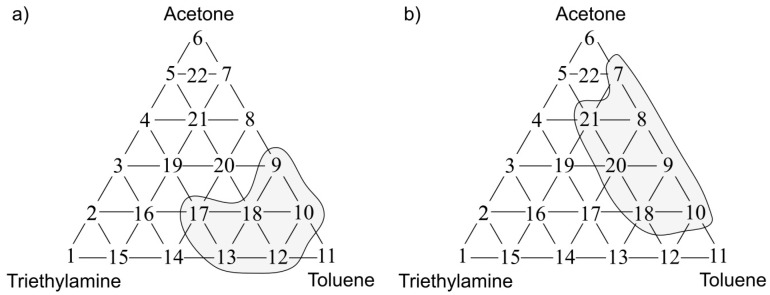
(**a**) Olfactory triangle for toluene-acetone-triethylamine mixture (set A) with indicated places of odour intensity amplification; (**b**) with indicated places of hedonic tone amplification. Analysis with PCR method.

**Figure 14:**
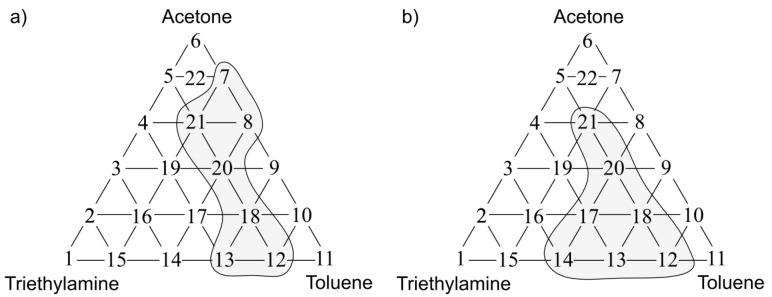
(**a**) Olfactory triangle for toluene-acetone-triethylamine mixture (set B) with indicated places of odour intensity amplification; (**b**) with indicated places of hedonic tone amplification. Analysis with PCR method.

**Figure 15:**
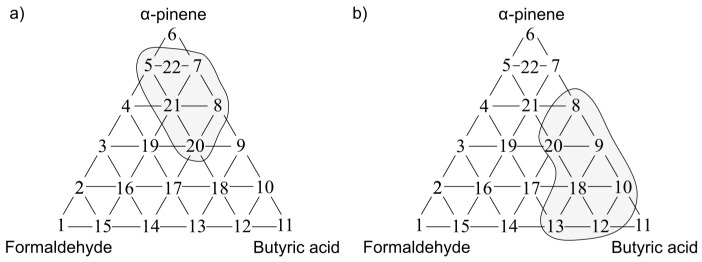
(**a**) Olfactory triangle for formaldehyde-butyric acid-pinene mixture (set C) with indicated places of odour intensity amplification; (**b**) with indicated places of hedonic tone amplification. Analysis with PCR method.

**Figure 16:**
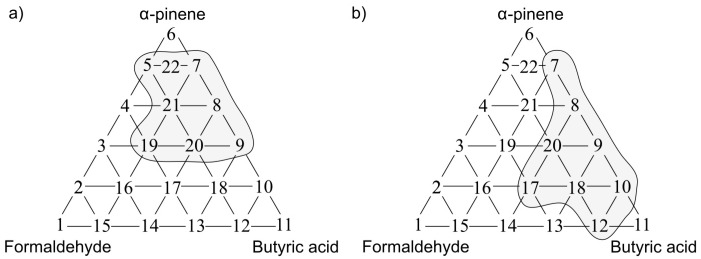
(**a**) Olfactory triangle for formaldehyde-butyric acid-pinene mixture (set D) with indicated places of odour intensity amplification; (**b**) with indicated places of hedonic tone amplification. Analysis with PCR method.

The following situations were observed when analogous investigation was performed using the PCR method as a tool for anticipation of odour interaction. Figure 13a presents the olfactory triangle for the toluene-acetone-triethylamine mixture (where odour intensity of the samples 1, 6, 11 equalled 1, set A) where one can observe six measurement points revealing amplification of odour intensity. The olfactory triangle for the same three-component mixture (Figure 13b) shows seven measurement points characterized by amplification of hedonic tone. Figure 14, Figure 15 and Figure 16 illustrate the olfactory triangles for the measurement sets B, C and D with indicated places of odour amplification. Table 4 contains the information about a number of the measurement points within the olfactory triangles representing the measurement sets A, B, C and D, at which odour amplification occurred. 

### 3.5. Comparison of Information about Odour Interactions Obtained Via Sensory Analysis and Electronic Nose Instrument

The aforementioned investigations, carried out by a group of assessors as well as using the electronic nose instrument and utilizing the PCR models, led to acquisition of the following information. Table 5 presents a number of the measurement points, for which both types of investigation indicated odour amplification. This table also includes the information about percentage of correct identifications obtained with both methods. Analysing Table 5 it can be observed that the level of correct identification of odour intensity amplification using the electronic nose and the PCR method was 80% for the measurement set A, 75% for the measurement set B, 80% for the set C and 75% for the set D. Analogously, the level of correct identification of hedonic tone amplification using the electronic nose and the PCR method was 67% for the measurement set A, 60% for the measurement set B, 57% for the set C and 73% for the set D. It is evident that in case of investigation of odour interactions employing the electronic nose coupled with PCR calibration method the correctness of identification of odour intensity amplification was higher than correctness of identification of hedonic tone amplification.

### 3.6. Evaluation of Constructed PCR Calibration Model

**Figure 17:**
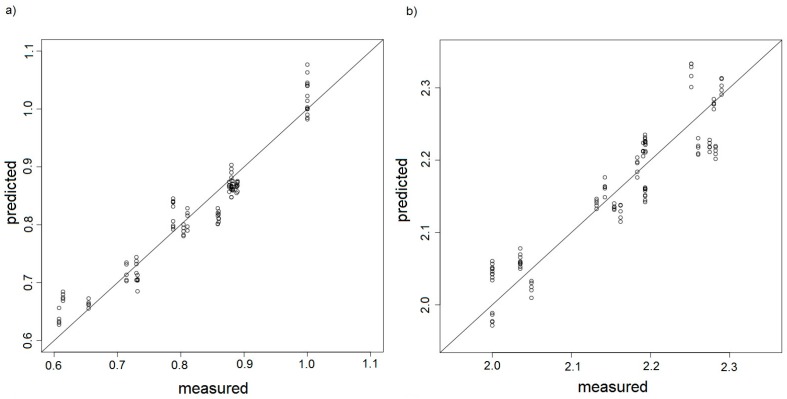

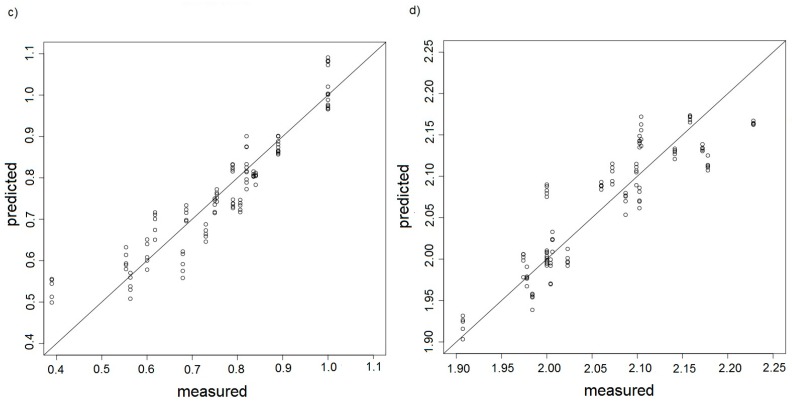
PCR model presented as predicted odour intensity versus odour intensity determined experimentally by a group of assessors, (**a**) measurement set A; (**b**) measurement set B; (**c**) measurement set C; (**d**) measurement set D.

The plots of predicted dependent variable (in this case odour intensity or hedonic tone determined with the PCR method) versus experimental variable (values of odour intensity or hedonic tone identified by a group of assessors) are the most frequent way of visual evaluation of the calibration models. The correlation plots of the PCR models were prepared only for the training set. They present fitting of the model to the training data. Figure 17a–d show the dependence between predicted odour intensity based on the PCR model and the intensity observed experimentally for the measurement sets A, B, C, D, respectively.

**Figure 18:**
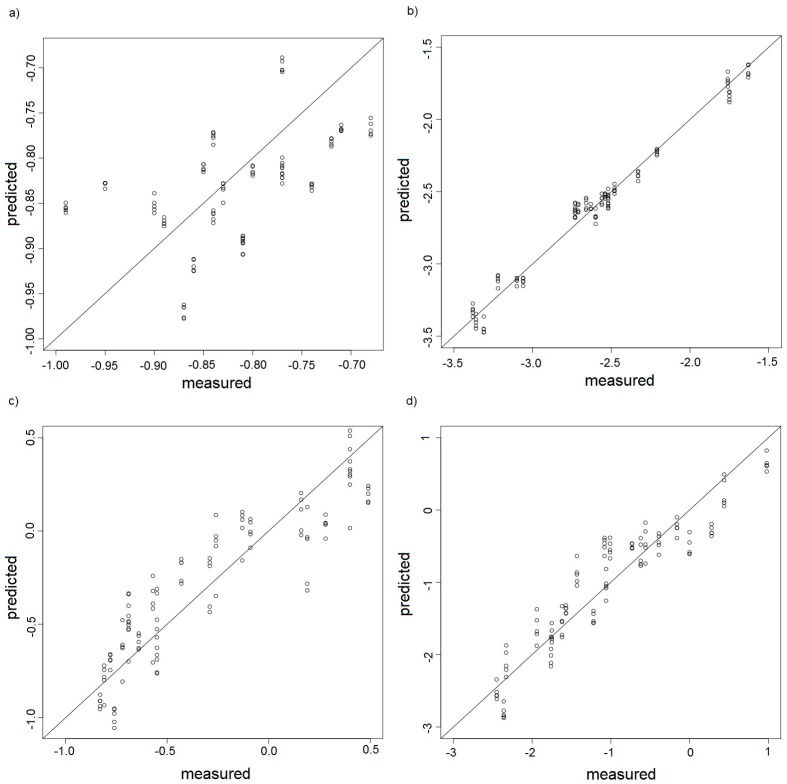
PCR model presented as predicted hedonic tone versus hedonic tone determined experimentally by a group of assessors, (**a**) measurement set A; (**b**) measurement set B; (**c**) measurement set C; (**d**) measurement set D.

It is easy to notice in these plots that in case of good calibrations models the samples of model and test sets should be distributed symmetrically along a straight of slope 1. Better calibration model was observed for the measurement sets A and C where odour intensity oscillated around 1. In case of the measurement sets B and C, for which intensity oscillated around 2, one can observe underestimated or overestimated points at the plots. Such situation can be an evidence that the measurement sensors comprising the electronic nose operated close to the upper limit of their detection abilities. Hence, these results incorporated in the PCR model could be burdened with additional errors. Figure 18a–d shows dependence between predicted hedonic tone based on the PCR model and the hedonic tone observed experimentally for the measurement sets A, B, C, D, respectively.

This time the plots of predicted value versus experimental value reveal much worse fitting. It is relatively easy to interpret as odour intensity is directly connected with concentration of particular odorant. Odorant concentration has a direct influence on a signal of the sensor being a part of the electronic nose instrument. In case of hedonic tone, although this quantity is also connected with odorant concentration, there is another factor, namely hedonic impact. This factor is relatively well-recognized by human but it is very difficult for a chemical sensor and requires a lot of experimental data as well as application of suitable data analysis such as neural networks or Kohennen network. However, the aim of the authors of this paper was to show that the PCR-type calibration model based on hidden variables was also capable of qualitative description of the odour interaction situations. Table 6 presents the values of root mean square error of prediction (RMSEP), number of optimum factors, for which the PCR model was constructed and coefficient of fitting to the optimum number of the PCR model factors. RMSEP can be used as a validation factor for the constructed calibration model.

In case of the data, for which predicted odour intensity was determined the average RMSEP was at the level of 0.03–0.06 and coefficient of determination for optimum number of the PCR model factors amounted 0.86–0.92. In case of the data, for which predicted hedonic tone was identified the average RMSEP was at the level of 0.07–0.34 and coefficient of determination for optimum number of the PCR model factors amounted 0.30–0.98. 

## 4. Conclusions

The paper proposes an application of an electronic nose as an instrumental tool consisting of semiconductor sensors and PID-type sensor to determine if it is possible to observe odour interaction phenomena for odorous three-component mixtures. The PCR calibration model was suggested for data analysis. The results, obtained by both a group of assessors as well as the electronic nose, revealed odour amplification in selected three-component mixtures. This effect was more evident for the mixtures, which were characterized by odour intensity at the level of ca. 2 as compared to the mixtures with odour intensity of around 1. It was also observed that the PCR model was more efficient upon determination and prediction of odour intensity than upon prediction of hedonic tone. Correctness of odour interactions determination with the electronic nose instrument and the PCR model was the level of 75–80% in case of odour intensity and 57–73% for hedonic tone.

The three-component mixtures subjected to investigation consisted of typical odorous compounds present at municipal landfills and sewage treatment plants. Selection of the three-component mixtures was random and the intention of the authors was to show that it is feasible to observe odour interactions with the instrumental tool and that this technique can be helpful and supplementary one with respect to the olfactometric techniques.

## Figures and Tables

**Table 1:** Characteristics of the investigated odorous substances.

Substance	Odour Type	Vapour Pressure (hPa)	Olfactory Threshold in the Gas Phase (ppm)
Toluene	Pleasant and characteristic	29	0.33–2.9
Acetone	Fruity, sweet	233	13–100
Triethylamine	Fish, pungent	72	0.000032–0.48
Formaldehyde	Pungent, stifling	1.4	0.5–1
Butyric acid	Rancid, odour of sweat	0.6	0.00019–0.001
α-Pinene	Pine, resinous	5	0.018

**Table 2:** Concentration range (C), odour intensity (I) and hedonic tone (HT) of the samples: toluene, acetone, triethylamine (set A and B) and formaldehyde, butyric acid, pinene (set C and D).

		Toluene	Acetone	Triethylamine	Formaldehyde	Butyric Acid	α-Pinene
**Set A**	C (ppm v/v)	0.06–0.6	60–600	0.15–1.5			
	I	0–1	0–1	0–1			
	HT	−0.8–0	−0.9–0	−0.9–0			
**Set B**	C (ppm v/v)	0.4–3.7	390–3900	0.5–5.5			
	I	0.8–2	0.75–2	0.25–2			
	HT	−1.6–−0.6	−1.75–−0.7	−1.8–−0.2			
**Set C**	C (ppm v/v)				50–540	0.5–4.7	0.01–0.13
	I				0–1	0–1	0–1
	HT				−0.8–0	−0.8–0	0–0.5
**Set D**	C (ppm v/v)				360–3600	1.5–14.5	0.05–0.5
	I				0.8–2	0–2	0.3–2
	HT				−1.6–−0.6	−1.6–0	0.15–1

**Table 3:** Comparison of experimental and theoretical values of olfactory threshold for aqueous solutions of toluene, acetone, trimethylamine, formaldehyde, butyric acid and pinene samples [60,61].

Substance	Experimental Olfactory Threshold (ppm v/v)	Theoretical Olfactory Threshold in Aqueous Solution (ppm v/v)
Toluene	0.1	0.04
Acetone	100	20–500
Triethylamine	0.4	0.4
Formaldehyde	81	60
Butyric acid	1.5	0.24
α-Pinene	0.03	0.14

**Table 4:** Number of measurement points for three-component mixtures of A, B, C, D sets where odour amplification phenomenon was observed using electronic nose instrument.

Set	Odour Intensity	Hedonic Tone
Set A	6	7
Set B	7	7
Set C	6	7
Set D	8	8

**Table 5:** Comparison of information obtained from sensory analysis and electronic nose measurements regarding occurrence of odour interactions in three-component mixtures-A, B, C and D sets.

Set	Odour Intensity		Hedonic Tone	
	Number	Percentage	Number	Percentage
Set A	4	80%	4	67%
Set B	6	75%	6	60%
Set C	4	80%	4	57%
Set D	6	75%	8	73%

**Table 6:** Values of root mean square error of prediction, optimum number of PCR model factors and coefficient of determination of model.

	Odour Intensity			Hedonic Tone		
Set	RMSEP	Number of Optimum Factors	R^2^	RMSEP	Number of Optimum Factors	R^2^
Set A	0.03	6	0.92	0.08	4	0.30
Set B	0.04	6	0.86	0.07	6	0.98
Set C	0.06	6	0.87	0.20	6	0.80
Set D	0.04	6	0.8	0.34	6	0.87
